# A Long Noncoding RNA ZEB1-AS1 Promotes Tumorigenesis and Predicts Poor Prognosis in Glioma

**DOI:** 10.3390/ijms17091431

**Published:** 2016-08-30

**Authors:** Qiao-Li Lv, Lei Hu, Shu-Hui Chen, Bao Sun, Meng-Long Fu, Chong-Zhen Qin, Qiang Qu, Gui-Hua Wang, Chen-Jie He, Hong-Hao Zhou

**Affiliations:** 1 Department of Clinical Pharmacology, Xiangya Hospital, Central South University, Changsha 410008, China; lvqiaoli2008@126.com (Q.-L.L.); hu773589905@163.com (L.H.); scy_csu2016@163.com (B.S.); m13397602527@163.com(M.-L.F.); hechenjie1307@163.com (C.-J.H.); 2 Institute of Clinical Pharmacology, Hunan Key Laboratory of Pharmacogenetics, Central South University, Changsha 410078, China; 3 Department of Oncology, Changsha Central Hospital, Changsha 410008, China; chenshuhui2008@126.com (S.-H.C.); zhuifenglang12@126.com (G.-H.W.); 4 Department of Pharmacy, The First Affiliated Hospital of Zhengzhou University, Central South University, Zhengzhou 450052, China; qcz0378@126.com; 5 Department of Pharmacy, Xiangya Hospital, Central South University, Changsha 410008, China; quqiang1983@sina.com

**Keywords:** long non-coding RNA, lncRNA ZEB1-AS1, glioma, prognostic biomarker, epithelial-mesenchymal transition

## Abstract

Emerging studies show that long noncoding RNAs (lncRNAs) have important roles in carcinogenesis. lncRNA ZEB1 antisense 1 (ZEB1-AS1) is a novel lncRNA, whose clinical significance, biological function, and underlying mechanism remains unclear in glioma. Here, we found that ZEB1-AS1 was highly expressed in glioma tissues, being closely related to clinical stage of glioma. Moreover, patients with high ZEB1-AS1 levels had poor prognoses, with the evidence provided by multivariate Cox regression analysis indicating that ZEB1-AS1 expression could serve as an independent prognostic factor in glioma patients. Functionally, silencing of ZEB1-AS1 could significantly inhibit cell proliferation, migration, and invasion, as well as promote apoptosis. Knockdown of ZEB1-AS1 significantly induced the G0/G1 phase arrest and correspondingly decreased the percentage of S phase cells. Further analysis indicated that ZEB1-AS1 could regulate the cell cycle by inhibiting the expression of G1/S transition key regulators, such as Cyclin D1 and CDK2. Furthermore, ZEB1-AS1 functioned as an important regulator of migration and invasion via activating epithelial to mesenchymal transition (EMT) through up-regulating the expression of ZEB1, MMP2, MMP9, N-cadherin, and Integrin-β1 as well as decreasing E-cadherin levels in the metastatic progression of glioma. Additionally, forced down-regulation of ZEB1-AS1 could dramatically promote apoptosis by increasing the expression level of Bax and reducing Bcl-2 expression in glioma. Taken together, our data suggest that ZEB1-AS1 may serve as a new prognostic biomarker and therapeutic target of glioma.

## 1. Introduction

Glioma accounts for the great majority of primary tumors in adult central nervous systems [1]. Despite advances in brain tumor therapy, consisting of surgical resection, chemotherapy, and radiation therapy, the prognosis of patients afflicted with this disease remains dismal, with the lowest five-year survival rate among all cancers [2,3] and a median survival of approximately 15 months [4,5]. Therefore, it is necessary to identify novel biomarkers specific to the stages of glioma or to the susceptibility of gliomas to anti-cancer agents, which may be helpful to develop more rational and effective therapies.

Long non-coding RNAs (LncRNAs) are commonly considered as a kind of non-coding RNA longer than 200 bases with no protein-coding capability [6]. Increasing evidence demonstrates that lncRNAs widely take part in the regulation of gene expression in various levels, including chromatin modification [7], transcription, and post-transcriptional processing involving many kinds of biological processes. In recent decades, it has been reported that aberrant expression of many lncRNAs is frequently observed in cancers involved in carcinogenesis and cancer progression which indicates that dysregulated lncRNAs probably could serve as novel biomarkers for early diagnosis, effective therapeutic targets, and prognosis prediction of malignant tumors. For instance, induction of lncRNA highly up-regulated in liver cancer (HULC), TCF7, and Zinc finger antisense 1 (ZFAS1) could promote tumorigenesis and metastasis of hepatocellular carcinoma (HCC) [8,9,10]. Overexpression of metastasis-associated lung adenocarcinoma transcript 1 (MALAT1), SBF2 antisense RNA 1 (SBF2-AS1), and Homo sapiens TatD DNase domain containing 1 (TATDN1) is capable of enhancing the ability of proliferation, metastasis, and invasion of lung cancer [11,12,13]. Up-regulation of colon cancer associated transcript 2 (CCAT2) and EPB41L4A-AS2 is associated with the prognosis of breast cancer [14,15]. High expression of HOXA11-AS and AB073614 is able to strengthen the proliferation and might serve as an unfavorable prognosis predictor for glioma [16,17], whereas lncRNA TSLC1 antisense RNA (TSLC1-AS1) has been characterized as a glioma suppressor and could serve as a biomarker and novel therapeutic target for glioma patients [18].

Recently, T Li and colleagues found that lncRNA ZEB1-AS1—a non-coding antisense transcript that originates from the promoters of ZEB1—was highly expressed in hepatocellular carcinoma, especially in metastatic liver tumor tissues, and related to poor outcome of HCC patients [19], with its similar role in esophageal squamous cell carcinoma (ESCC) having also been observed by Wang et al. [20]. However, the implication of ZEB1-AS1 in glioma was still unclear. In our study, we detected the mRNA expression level of ZEB1-AS1 in glioma tissues as well as in normal brain tissues, and explored the relationship of ZEB1-AS1 with clinical characteristics and prognosis of glioma patients. Moreover, using ZEB1-AS1-specific siRNA, we further investigated the effects as well as the potential molecular mechanism of ZEB1-AS1 downregulation involved in proliferation, apoptosis, migration and invasion of U87 and U251 cells in vitro.

## 2. Results

### 2.1. Expression of lncRNA ZEB1-AS1 is Up-Regulated in Glioma Tissues

**Figure 1:**
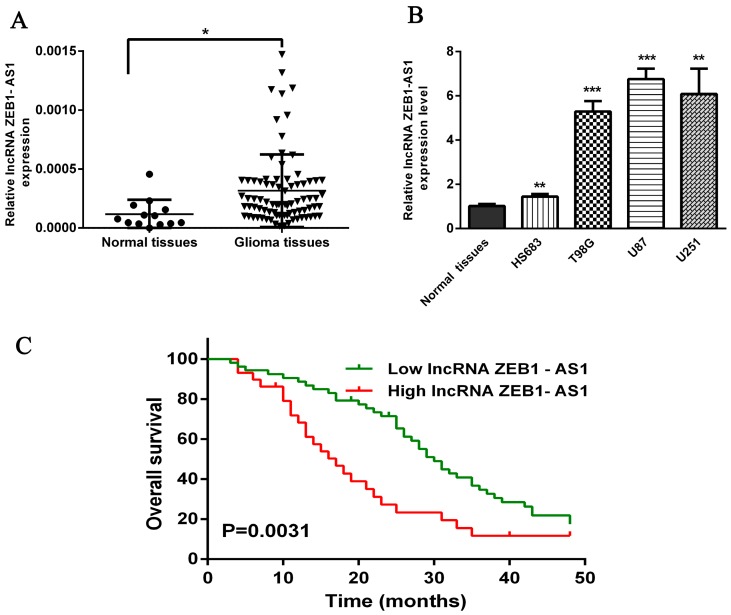
Relative expression of the long noncoding RNA (lncRNA) ZEB1-AS1 in glioma tissues and its clinical significance. (**A**) qRT–PCR analysis of ZEB1-AS1 expression level in 13 normal brain tissues and 82 glioma tissues; (**B**) The expression of ZEB1-AS1 in glioma cell lines and normal brain tissues; (**C**) Patients with high levels of ZEB1-AS1 expression were correlated with a poor overall survival in glioma (*p* = 0.0031, Kaplan-Meier method). * *p* < 0.05, ** *p* < 0.01, *** *p* < 0.001.

It was reported that lncRNA ZEB1-AS1 was greatly up-regulated in human hepatocellular carcinoma and in esophageal squamous cell carcinoma compared with matched normal tissues [19,20]. To investigate the implication of this lncRNA in glioma, we detected the expression levels of ZEB1-AS1 in glioma tissues by qRT-PCR, finding that ZEB1-AS1 expression was statistically up-regulated in glioma tissues compared with the normal brain tissues (Figure 1A, *p* < 0.05). Furthermore, we measured the expression levels of ZEB1-AS1 in glioblastoma cell lines and normal brain tissues with qRT-PCR, finding that they were significantly higher in three high-degree glioblastoma cell lines (T98G, U87, U251) than in the low-degree glioma cell line (HS683) and normal brain tissues (Figure 1B).

### 2.2. Relationship of lncRNA ZEB1-AS1 Expression with Clinicopathological Features of Glioma Patients

To further elucidate the significance of ZEB1-AS1 in glioma, we calculated the correlation of ZEB1-AS1 expression with clinicopathological features of 82 glioma patients (as shown in Table 1), finding that highly expressed ZEB1-AS1 was greatly related to tumor grade of glioma (*p* = 0.018). However, no association of ZEB1-AS1 expression with age, gender, and tumor location (*p* = 0.267, 0.770, and 0.517, respectively) was detected. Thus, we speculated that ZEB1-AS1 might have important implications for the progression of glioma.

### 2.3. High Expression Levels of lncRNA ZEB1-AS1 were Significantly Related to Reduced Overall Survival of Glioma Patients

In order to further determine the prognostic significance of lncRNA ZEB1-AS1 in glioma, we examined the relationship of ZEB1-AS1 levels with overall survivor (OS) rates through Kaplan-Meier analysis and log-rank test in 82 glioma cases, discovering that high ZEB1-AS1 expression might predict a poor overall survival (Figure 1C, Hazard Ratio (HR) = 2.055, 95%Confidence Interval (CI): 1.384–4.462, *p* = 0.0031).

### 2.4. lncRNA ZEB1-AS1 Expression was a Potentially Independent Prognostic Marker for Glioma Patients

Results of univariate Cox regression analysis revealed that both ZEB1-AS1 overexpression (HR = 2.119, 95% CI: 1.265–3.551, *p* = 0.004) and the clinical stage (HR = 2.141, 95% CI: 1.286–3.563, *p* = 0.003) were prime variables for glioma prognosis (Table 2). Furthermore, with age, gender, and tumor location as covariates, multivariate Cox regression analysis indicated that highly expressed ZEB1-AS1 (HR = 1.885, 95% CI: 1.068–3.326, *p* = 0.029) and the clinical stage (HR = 1.791, 95% CI: 1.016–3.158, *p* = 0.044) were independent prognostic factors for overall survival of glioma prognosis (Table 2).

### 2.5. siRNA-Induced lncRNA ZEB1-AS1 Silence in U87 and U251 Cells

**Figure 2:**
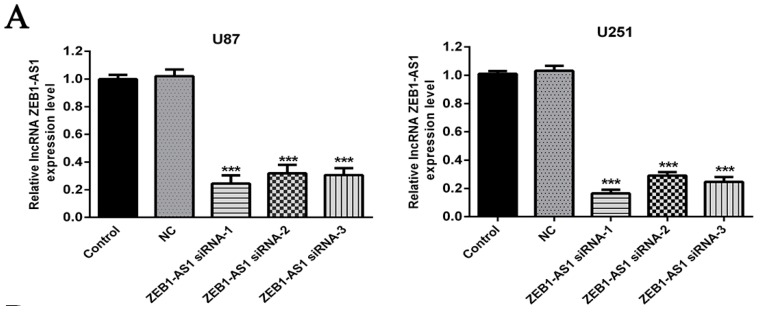

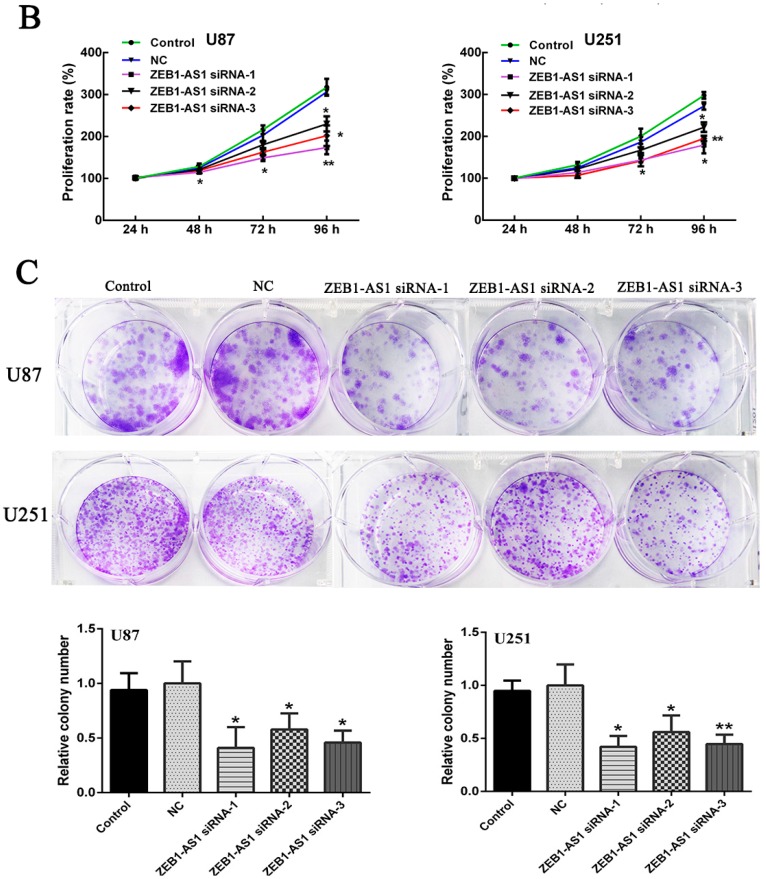
Knockdown of lncRNA ZEB1-AS1 inhibited the proliferation of glioma cell lines. (**A**) Silence of lncRNA ZEB1-AS1 expression in glioma cell lines by ZEB1-AS1-specific siRNAs; (**B**) CellTiter 96 aqueous one solution reagent (MTS) assay indicated that all of the three ZEB1-AS1 siRNAs could significantly suppress the proliferation abilities of U87 and U251 cells compared with negative control (NC) groups at 96 h; (**C**) Colony formation assay revealed that the silencing of ZEB1-AS1 greatly reduced the number of colonies of the U87 and U251 cells in comparison with the NC groups. * *p* < 0.05, ** *p* < 0.01, *** *p* < 0.001.

To further explore whether ZEB1-AS1 was associated with the progression of glioma, function studies were conducted in vitro. Because of the high expression of ZEB1-AS1 in glioma tissues and cell lines, three ZEB1-AS1-specific siRNAs were adopted to knock down ZEB1-AS1 expression in U87 and U251 cells, aiming to further examine the role of ZEB1-AS1 in glioma. After transfection, the expression of ZEB1-AS1 was determined using qRT-PCR. As shown in Figure 2A, all of the three ZEB1-AS1-siRNAs could silence ZEB1-AS1 expression effectively in U87 and U251 cells, compared with the non-specific siRNA (NC) groups.

### 2.6. Silencing of lncRNA ZEB1-AS1 Inhibited U87 and U251 Cells Proliferation

Cell proliferation was examined via MTS and colony formation assays after the knockdown of ZEB1-AS1 in U87 and U251 cells by siRNAs. MTS assay suggested that all of the three ZEB1-AS1-siRNAs could significantly decrease the proliferation abilities of U87 and U251 cells in contrast with negative control (NC) groups (Figure 2B) in a time-dependent manner. Additionally, colony formation assay demonstrated that the number of U87 and U251 cell colonies (Figure 2C) was significantly reduced by the knockdown of ZEB1-AS1 compared with the NC groups. According to Figure 2A, ZEB1-AS1 siRNA-1 showed the best interference efficiency, thus it was chosen for subsequent research.

**Figure 3:**
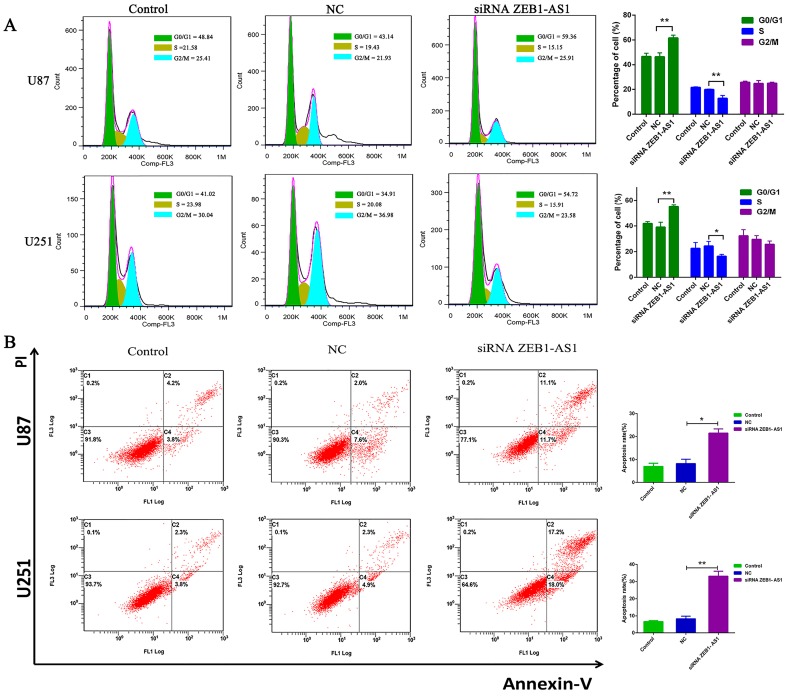
Effects of lncRNA ZEB1-AS1 on glioma cell cycle and apoptosis were analyzed by flow cytometry. (**A**) Cell cycle analysis showed that the silencing of ZEB1-AS1 greatly induced the G0/G1 phase arrest and correspondingly decreased the percentage of S phase cells; (**B**) Annexin V/PI (propidium iodide) staining and flow cytometry analysis showed that ZEB1-AS1 knockdown in U87 and U251 cells dramatically enhanced the cell apoptosis rate compared with NC groups. * *p* < 0.05, ** *p* < 0.01.

To investigate the mechanisms underlying growth suppression after ZEB1-AS1 knockdown, we further examined its effect on cell cycles of U87 and U251 cells by propidium iodide (PI) staining and flow cytometry. Flow cytometric analysis revealed that knock-down of ZEB1-AS1 dramatically caused the G0/G1 phase arrest and correspondingly decreased the percentage of S phase cells (Figure 3A, *p* < 0.05 for U87, *p* < 0.05 for U251).

Collectively, these results suggested that the silencing of ZEB1-AS1 could inhibit tumor cell proliferation and delay the progress of tumor by intervening cell mitosis and inducing cell cycle arrest.

### 2.7. Silencing of lncRNA ZEB1-AS1 Promoted U87 and U251 Cells Apoptosis

As defects in apoptosis could lead to the occurrence of tumor development, we subsequently explored whether knockdown of ZEB1-AS1 greatly increased the number of apoptosis glioma cells. Flow cytometry analysis revealed that knocking down ZEB1-AS1 in U87 and U251 cells significantly enhanced the cell apoptosis rate in comparison with NC groups (Figure 3B, *p* < 0.05 for U87, *p* < 0.05 for U251), indicating the crucial role of ZEB1-AS1 in the apoptosis regulation of human glioma cells.

### 2.8. Silencing of lncRNA ZEB1-AS1 Inhibited the Migration and Invasion Abilities of U87 and U251 Cell Lines

**Figure 4:**
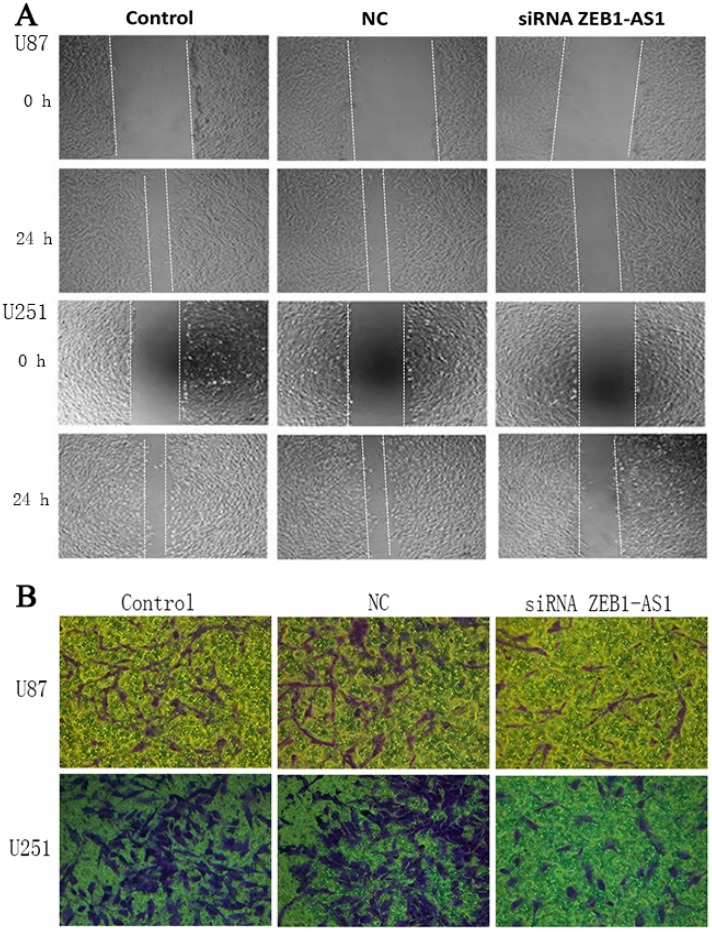
Silencing of lncRNA ZEB1-AS1 inhibited the migration and invasion ability of glioma cells. (**A**) The wound healing assay demonstrated that ZEB1-AS1 knockdown in U87 and U251 cells could significantly inhibit cell migration compared with the NC groups; (**B**) For the transwell invasion assay, most of the U87 and U251 cells invaded from the top chambers to the bottom chambers in the control and NC groups, but not in the ZEB1-AS1-siRNA groups. Scale bar: 100 μm.

The changes of migration and invasion capabilities of U87 and U251 cells were monitored at 24 h after transfection of ZEB1-AS1-specific siRNA. As indicated in Figure 4, the inhibition of ZEB1-AS1 could significantly decrease the migration and invasion ability of human glioma cells compared with controls.

### 2.9. Signaling Pathways Relative to Proliferation, Migration, and Invasion of U87 and U251 Cells

**Figure 5:**
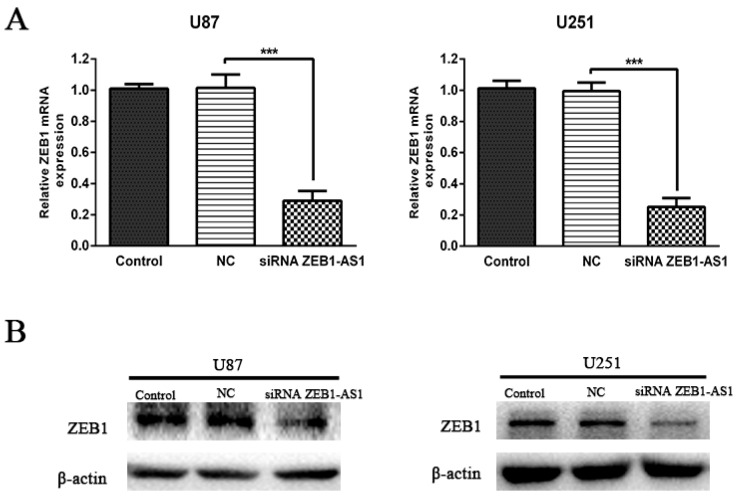
Knockdown of lncRNA ZEB1-AS1 decreased the expression of ZEB1. (**A**,**B**) Levels of ZEB1 mRNA and protein were detected by qRT-PCR and western blot after ZEB1-AS1 knockdown in U87 and U251 cell lines. *** *p* < 0.001.

Recent studies have demonstrated that upstream antisense transcription may function as a regulator of corresponding gene expression. ZEB1-AS1 is an antisense lncRNA, transcribed from the antisense chain of the protein coding ZEB1 gene. To identify whether ZEB1-AS1 could modulate the expression of ZEB1, we first knocked down ZEB1-AS1 expression in U87 and U251 cells by transfecting ZEB1-AS1-specific siRNA. The results demonstrated that knockdown of ZEB1-AS1 could significantly decrease the expression of ZEB1 both at mRNA and protein levels (Figure 5A,B).

**Figure 6:**
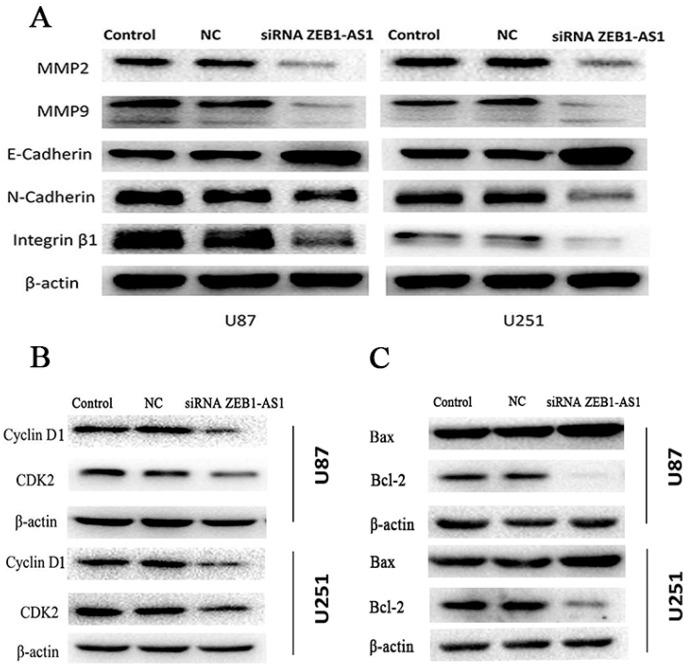
Protein levels of cell cycle, EMT, and apoptosis markers after lncRNA ZEB1-AS1 knockdown. (**A**–**C**) The expression of Cyclin D1, CDK2, MMP2, MMP9, N-cadherin, Integrin-β1, and Bcl-2 significantly decreased, while the expression of E-cadherin and Bax was elevated in the knockdown of the ZEB1-AS1 group compared with the NC groups, both in U87 and U251 cell lines.

Due to ZEB1 being a crucial transcription factor for regulating epithelial to mesenchymal transition (EMT), we suspected that ZEB1-AS1 might promote glioma cell migration and invasion by regulation ZEB1-EMT axis. Western blot was conducted to detect the protein expression of well-recognized molecules related to the invasion and metastasis of tumors, such as MMP2, MMP9, E-cadherin, N-cadherin, and β1-Integrin. As shown in Figure 6A, compared to NC groups, the expression of MMP2, MMP9, N-cadherin, and Integrin-β1 in U87 and U251 cells diminished, while the expression of E-cadherin was elevated significantly after ZEB1-AS1-siRNA transfection.

Moreover, we investigated the protein levels of G1/S transition key regulators, such as Cyclin D1 and CDK2. As shown in Figure 6B, Cyclin D1 and CDK2 protein expression was significantly lower in the ZEB1-AS1 knockdown group than the NC groups, both in U87 and U251 cell lines. Additionally, apoptosis markers were also examined, as shown in Figure 6C, the expression levels of Bax were dramatically increased, while Bcl-2 expression was greatly reduced after ZEB1-AS1-siRNA transfection, demonstrating that the overexpression of ZEB1-AS1 probably played a crucial role in the regulation of the proliferation, apoptosis, migration, and invasion of human glioma cells.

## 3. Discussion

With the impressive technology advancements, a great number of lncRNAs, initially considered as transcriptional noise [21,22,23], have been demonstrated to be dysregulated in wide spectrum of human diseases, such as diabetes [24,25,26,27,28,29], kidney dysfunction [30], cardiovascular diseases [31], and especially malignant tumors [32,33,34]. Increasing reports of aberrant expression of lncRNAs across numerous cancer types suggested that some lncRNAs could promote the formation and development of tumors [35,36,37].

In this study, we first discovered that the expression of lncRNA ZEB1-AS1 significantly increased in glioma tissues. Further, we explored the association between ZEB1-AS1 expression and clinical characteristics of glioma patients, finding that its expression was significantly related with the clinical stage, suggesting its crucial role in the progress of glioma. Moreover, we calculated the correlation of ZEB1-AS1 expression with overall survival, observing that patients with highly expressed ZEB1-AS1 had a decreased rate of overall survival. More importantly, multivariate Cox regression analysis provided evidence that both clinical stage and ZEB1-AS1 expression could serve as independent poor prognostic factors in patients with glioma, which was compatible with the research results by Li et al. and Wang et al. that up-regulated expression of ZEB1-AS1 promoted tumor metastasis and predicted poor prognosis in HCC and ESCC patients [19,20].

Additionally, function and mechanism research was conducted in cell models to find out the precise role of ZEB1-AS1 in glioma. Li et al. provided evidence that highly expressed ZEB1-AS1 could enhance the proliferation, migration, and invasion ability of HCC cells both in vitro and in vivo [19]. In our study, we observed that the silencing of ZEB1-AS1 could inhibit cell cycle, proliferation, migration, and invasion, and promote apoptosis of glioma cells.

Li et al. discovered in HCC cells that overexpression of ZEB1-AS1, greatly inducing the expression and activity of ZEB1, could lead to a decreased protein level of E-cadherin, an epithelial marker, as well as an increased expression of mesenchymal markers, such as Vimentin, N-cadherin, and Fibronectin [19]. Correspondingly, we not only found similar results in that knockdown of ZEB1-AS1 caused up-regulated E-cadherin and down-regulated N-cadherin, but also detected that Integrin-β1, MMP2, and MMP9 were markedly reduced in the present study. Since the ZEB1-AS1 gene is located at the promoter of ZEB1, an important transcription factor participating in EMT, we speculate that lncRNA ZEB1-AS1 could affect the cell migration and invasion through the ZEB1-EMT pathway in glioma patients.

**Figure 7:**
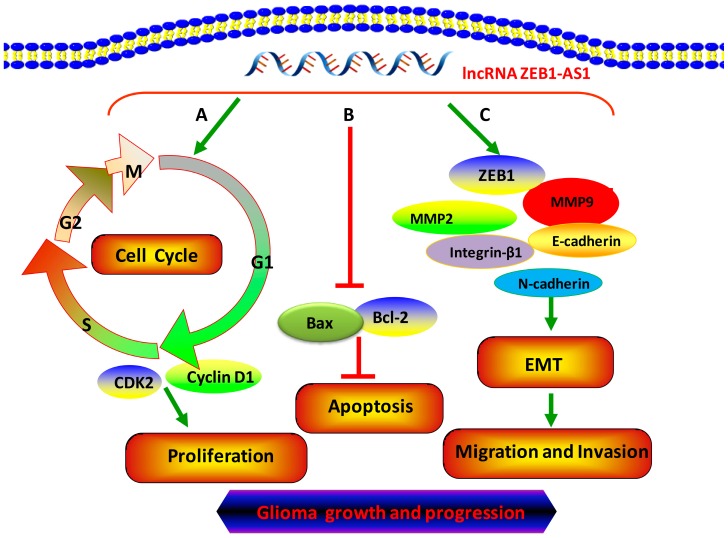
lncRNA ZEB1-AS1 was a key regulator of glioma proliferation, apoptosis, migration, and invasion. (**A**) ZEB1-AS1 promoted the proliferation by significantly inducing the G0/G1 phase arrest and correspondingly decreasing the percentage of S phase in cell cycle; (**B**) ZEB1-AS1 inhibited the apoptosis of glioma cells; (**C**) ZEB1-AS1 activated EMT through up-regulating the expression of MMP2, MMP9, N-cadherin, and Integrin-β1 as well as decreasing E-cadherin levels and accelerating the migration and invasion of glioma cells.

Apoptosis is a crucial factor in the transformation and progression of malignant tumors. Bcl-2 family members, mainly including the upstream pro-apoptotic member Bax and downstream anti-apoptotic member Bcl-2, play an essential role in the process of cancer cell apoptosis. In our study, we found that silencing ZEB1-AS1 could dramatically promote cell apoptosis partly by increasing the expression of Bax and inhibiting the expression of Bcl-2 in both U87 and U251 cells. We also firstly discovered that the protein levels of G1/S transition key regulators, cyclin D1 and CDK2, were significantly decreased when ZEB1-AS1 was silenced. Collectively, ZEB1-AS1 plays a vital role in regulating the proliferation and progression of glioma cells (Figure 7).

Additionally, our laboratory study found that ZEB1-AS1 was involved in temozolomide (TMZ) resistance. All of these findings demonstrated that the overexpression of ZEB1-AS1 played a pivotal role in the modulation of glioma progression. However, the manner in which ZEB1-AS1 regulates the genes that are related to the proliferation, migration, invasion, and apoptosis of glioma cell lines was not clarified in our study and further studies will be performed in the future.

## 4. Materials and Methods

### 4.1. Tissue Samples

Eighty-two glioma samples were acquired from patients who experienced surgery resection from November 2010 to June 2013 at the Department of Neurosurgery, First Affiliated Hospital of Nanchang University (Nanchang, China). Thirteen normal brain tissues from patients with cerebral trauma/epilepsy surgery were used as controls. Samples were instantly frozen in liquid nitrogen and stored at −80 °C until the extraction of total RNA. Patients who had ever undergone radiotherapy, chemotherapy, and surgery were excluded from this study. All of the patients experienced a follow-up period lasting 48 months since the surgery. The protocol used in this study was approved by the Research Ethics Committee of First Affiliated Hospital of Nanchang University (Ethical Approval No. 2010-015; Date: 12 March 2010), with written informed consent obtained from every participant.

### 4.2. Cell Lines and Culture

The human glioma HS683, T98G, U87, and U251 cell lines were obtained from the American Type Culture Collection, cultured in Dulbecco’s modified Eagle’s medium (DMEM) medium with 10% fetal bovine serum (FBS, GIBCO, Carlsbad, CA, USA). All cells were maintained at 37 °C in a humidified atmosphere of 5.0% CO_2_.

### 4.3. Silencing of ZEB1-AS1 by siRNA

Three ZEB1-AS1-specific siRNAs were synthesized by Ribobio (Guangzhou, China). A non-specific scramble siRNA sequence was used as negative control (NC). The siRNAs were transiently transfected into U87 and U251 cells using Lipofecamine^®^ RNAiMAX Transfection Reagent (Invitrogen, Carlsbad, CA, USA) according to the manufacturer’s directions. Assays were conducted at 48 h after transfection.

### 4.4. RNA Isolation and Real-Time PCR

Total RNA was isolated from tissue samples or cultured cells using TRIzol reagent (Invitrogen, Carlsbad, CA, USA) according to the manufacturer’s instructions. cDNA was generated by reverse transcription of 1 μg of total RNA using Thermo Scientific RevertAid First Strand cDNA Synthesis Kit (Thermo Fisher Scientifi, Wilmington, DE, USA). qRT-PCR was conducted using SYBR Green real-time PCR Kit (Takara, Dalian, China) on a LightCycler480 (Roche, San Francisco, CA, USA) to detect the expression of lncRNA ZEB1-AS1, with GAPDH as a normalizing control. The primers for ZEB1-AS1 were 5′-AACCTTGTTGCTAGGGACCG-3′ and 5′-AGTCACTTCCCATCCCGGTT-3′; the primers for GAPDH were 5′-CCCATCACCATCTTCCAGGAG-3′ and 5′-GTTGTCATGGATGACCTTGGC-3′.

### 4.5. Western Blotting

Western blotting was performed as described in Zhang et al.’s research [38]. Anti-MMP2, anti-MMP9, anti-Integrinβ1, anti-ZEB1, anti-Bax, anti-Bcl-2, anti-Cyclin D1, and anti-CDK2 antibodies were purchased from CST Biotechnology (Boston, MA, USA). Antibodies against E-Cadherin and N-Cadherin were obtained from Santa Cruz Biotechnology (Santa Cruz, CA, USA) Antibody to β-actin was bought from Sigma and used as a loading control.

### 4.6. Cell Proliferation Assay

Cell proliferation was determined using MTS (CellTiter 96 aqueous one solution reagent, Promega, Madison, WI, USA) according to the manufacturer’s directions. In Brief, cells were cultured into 96-well plates at a density of 3 × 10^3^ cells in 200 μL medium per well. At the indicated time point, 10 μL of MTS diluted in 90 μL of culture medium was added into each well and incubated at 37 °C for 30 min. The absorbance at 490 nm was detected on a microplate reader (Bio-Rad Laboratories, Inc., Hercules, CA, USA). All experiments were carried out in sextuplicate and repeated three times.

### 4.7. Colony Formation Assay

Both non-transfected and transfected U87 and U251 cells were seeded in six-well plates at the density of 500 cells/well. After approximately 12 days, the cells were fixed with 4% paraformaldehyde for 30 min, and stained with 0.1% crystal violet (Beyotime Institute of Biotechnology, Shanghai, China) for 30 min. Next, the plates were washed mildly with phosphate-buffered saline (PBS) before being air-dried, and the stained colonies were photographed using a high-resolution camera. The experiments were performed at least in triplicate.

### 4.8. Flow Cytometry Analysis of Cell Cycle and Apoptosis

For cell cycle analysis, transfected U87 and U251 cells were plated in six-well plates, harvested by trypsinization after 48 h, and fixed with ice-cold 70% ethanol at −20 °C overnight. After being centrifuged and washed with ice PBS, the cells were then incubated in 300 μL staining buffer, 10 μL propidium iodide (PI), and 5 uL RNase (Beyotime Institute of Biotechnology, Shanghai, China) for 20 min at room temperature in the dark. Cells were analyzed by FC500 flow cytometry (Beckman Coulter, Bethesda, MA, USA). We calculated and compared the percentage of the cells in the G0/G1, S, and G2/M phases with the FlowJo software (version 7.6.5; Treestar, OR, USA).

Cell apoptosis was detected as Peng et al. described. The apoptosis rates were measured using Annexin V-FITC/PI Apoptosis Detection Kit (BD Biosciences, San Jose, CA, USA) according to the directions.

### 4.9. Wound Healing Assay

U87 and U251 cells were seeded in six-well plates for 24 h before being transfected with ZEB1-AS1-siRNA or NC. Forty-eight hours later, when the cell reached approximately 80%–90% density, an identical wound was made across the center of the well using a 200 μL plastic pipette tip. The remaining cells were washed with PBS for three times and then cultured in a medium containing 2% FBS. At 0 and 24 h after wounding, the size of wound was photographed using a microscope. Three separate experiments were carried out.

### 4.10. Transwell Invasion Assay

Cell invasion assays were conducted using transwell inserts containing an 8 μm pore size polycarbonate membrane (Corning, Tewksbury, MA, USA), with a thin layer of Matrigel (BD Bioscience, San Jose, CA, USA). The lower space was filled up using medium with 10% FBS. Transfected and non-transfected U87 and U251 cells were seeded to the upper part at a density of 1.0 × 10^5^ cells/mL in 0.2 mL of 1% FBS medium. After the incubation at 37 °C for 24 h, the inserts were taken out and washed mildly with PBS for a total of three times. The cells and Matrigel on the upper layer of the polycarbonate membrane were wiped gently with cotton swabs, then the membrane was fixed with 4% paraformaldehyde for 10 min and stained with crystal violet for 10 min. Next, the stained inserts were washed softly with PBS for a total of three times. Cells in randomly selected fields were photographed using a light microscope.

### 4.11. Statistical Analysis

SPSS 19.0 software system (IBM, SPSS, Chicago, IL, USA) was used for statistical analysis. All values were described as mean ± standard deviation (S.D.). The Student’s *t*-test was used for comparisons between groups. A χ-square test was applied to determine the association of ZEB1-AS1 levels with clinicopathologic features. Kaplan-Meier method was used to calculate the overall survival (OS) rates, and log-rank test was performed to calculate the difference of OS between groups with high and low ZEB1-AS1 expression. A Cox regression analysis was adopted to assess the prognostic factors. Differences were deemed statistically significant at *p* < 0.05.

## 5. Conclusions

Collectively, to our best knowledge, this is the first study showing that lncRNA ZEB1-AS1 is highly expressed in glioma tissues and its overexpression may be indicative of higher risks for brain cancer progression and poor overall survival rate, thereby suggesting its usage as an underlying prognostic marker and a new potential therapeutic target for the treatment of human gliomas.

## Figures and Tables

**Table 1:** Correlation between lncRNA ZEB1-AS1 expression and clinicopathological features of glioma patients.

Clinical Characteristic	No. of Patients	NO. of Patients		*p-*Value
		High Expression (29)	Low Expression (53)	
Age (year)				
<45	35	10	25	0.2668
≥45	47	19	28	
Sex				
Male	52	19	33	0.770
Female	30	10	20	
Clinical Stage				
Low Grades I–II	37	8	29	0.0183 *
High Grades III–IV	45	21	24	
Tumor Location				
Frontal	26	6	20	0.5170
Parietal	6	2	4	
Occipital	15	6	9	
Temporal	14	5	9	
Others	21	10	11	

* The values had statistically significant differences.

**Table 2:** Univariate and multivariate analyses of overall survivor (OS) rates in 82 glioma patients by Cox regression analysis.

Variable	Univariate Analysis	*p*-Value	Multivariate Analysis	*p*-Value
	HR (95% CI)		HR (95% CI)	
Age(<45 vs. ≥45 years)	1.030 (0.631–1.680)	0.907	1.085 (0.599–1.964)	0.788
Sex (female vs. male)	1.169 (0.709–1.928)	0.541	1.076 (0.638–1.818)	0.783
Clinical Stage (III–IV vs. I–II)	2.141 (1.286–3.563)	0.003 **	1.791 (1.016–3.158)	0.044 *
Tumor Location		0.636		0.848
Parietal vs. Frontal	0.806 (0.275–2.359)	0.694	0.969 (0.308–3.044)	0.957
Occipital vs. Frontal	1.117 (0.546–2.287)	0.762	1.080 (0.474–2.461)	0.855
Temporal vs. Frontal	1.625 (0.806–3.279)	0.175	1.408 (0.687–2.884)	0.350
Others vs. Frontal	1.044 (0.541–2.015)	0.899	0.904 (0.420–1.947)	0.796
ZEB1-AS1 Expression (high vs. low)	2.119 (1.265–3.551)	0.004 **	1.885 (1.068–3.326)	0.029 *

*; ** The values had statistically significant differences.
